# Air Pollution Metric Analysis While Determining Susceptible Periods of Pregnancy for Low Birth Weight

**DOI:** 10.1155/2013/387452

**Published:** 2013-01-30

**Authors:** Joshua L. Warren, Montserrat Fuentes, Amy H. Herring, Peter H. Langlois

**Affiliations:** ^1^Department of Biostatistics, University of North Carolina at Chapel Hill, Chapel Hill, NC 27599-7420, USA; ^2^Department of Statistics, North Carolina State University, Raleigh, NC 27695-8203, USA; ^3^Birth Defects Epidemiology and Surveillance Branch, Texas Department of State Health Services, Austin, TX 78714-9347, USA

## Abstract

Multiple metrics to characterize air pollution are available for use in environmental health analyses in addition to the standard Air Quality System (AQS) pollution monitoring data. These metrics have complete spatial-temporal coverage across a domain and are therefore crucial in calculating pollution exposures in geographic areas where AQS monitors are not present. We investigate the impact that two of these metrics, output from a deterministic chemistry model (CMAQ) and from a spatial-temporal downscaler statistical model which combines information from AQS and CMAQ (DS), have on risk assessment. Using each metric, we analyze ambient ozone's effect on low birth weight utilizing a Bayesian temporal probit regression model. Weekly windows of susceptibility are identified and analyzed jointly for all births in a subdomain of Texas, 2001–2004, and results from the different pollution metrics are compared. Increased exposures during weeks 20–23 of the pregnancy are identified as being associated with low birth weight by the DS metric. Use of the CMAQ output alone results in increased variability of the final risk assessment estimates, while calibrating the CMAQ through use of the DS metric provides results more closely resembling those of the AQS. The AQS data are still preferred when available.

## 1. Introduction

 Low birth weight, defined as less than 2,500 grams (g) at birth, is associated with immediate and long-term health effects, including death. Low birth weight affects around 8% of all births in the United States (US) with over two-thirds of those cases associated with premature birth [1]. For term births who result in low birth weight, fetal growth restriction is thought to be the major contributing factor, associated with a number of factors including smoking during the pregnancy, alcohol/drug abuse, birth defects, and certain socioeconomic factors [2–4]. Long-term health effects of low birth weight include type-2 diabetes, high blood pressure, heart disease, hearing/vision problems, and intellectual disabilities.

Previous ambient air pollution/birth weight epidemiologic studies have focused on low birth weight as a binary variable as well as working with continuous birth weight directly. Common analyses involve calculating pollution exposures based on active pollution monitors near the residence at delivery of the mother. Trimester averages are the most common time period of interest in these studies [5–12], while some studies have incorporated monthly exposure averages throughout the pregnancy [13, 14]. A recent literature review by Šrám et al. [15] concluded that for birth weight, there was a need for future studies “to clarify the most vulnerable periods of pregnancy and the role of individual pollutants.” In this paper, we thoroughly investigate these vulnerable periods in terms of ambient ozone exposure.

Previous studies have examined a number of pollutants and time frames with varying results. Carbon monoxide exposure during multiple periods of the pregnancy was shown to adversely affect the birth weight of the child in multiple studies [5, 7–9, 11], with most of the results indicating the first trimester as the most susceptible time. Sulfur dioxide was also consistently found to decrease birth weight in multiple studies during various trimesters and months of pregnancy [6, 8, 9, 12, 13]. A number of studies have also uncovered an adverse association between total suspended particles and birth weight [6, 8, 12]. The relationship between birth weight and ozone exposure is less clear.

In this paper, we utilize a Bayesian statistical model for low birth weight, which has the ability to more accurately identify critical periods during the pregnancy where increased exposure to ambient ozone concentrations significantly increases the probability of low birth weight of the child. This model was originally developed for a preterm birth analysis by Warren et al. [16]. We allow for a more continuous form of pollution exposure throughout the pregnancy than typically considered in the low birth weight context through the use of weekly ambient averages. Working in the Bayesian setting allows us to properly characterize the uncertainty in our model parameters while also helping to control the multicollinearity introduced by jointly considering weekly effects in the model.

We begin the analysis by assigning ozone pollution exposures to each woman in a subdomain of Texas, based on residence at delivery, during each week of the pregnancy for three different pollution metrics. These metrics include the standard Air Quality System (AQS) data, the Community Multiscale Air Quality (CMAQ) chemistry model output, and the newly developed downscaler (DS) pollution model output. The AQS data are the most commonly used in epidemiologic studies and represent observed monitoring data across the state, while the CMAQ output is based on a deterministic model which relies on meteorological input and pollutant specific chemistry expertise. The DS process calibrates the CMAQ output by statistically combining it with the AQS data in order to provide estimates of the AQS observations in regions lacking spatial-temporal monitor coverage. We investigate the use of the CMAQ and DS metrics with respect to risk assessment estimation and compare their results with results seen using the AQS data.

This metric comparison analysis will help to inform future studies which may require the use of alternative pollution output in rural or undeveloped geographical regions of interest. The alternate pollution metrics have complete spatial-temporal coverage over a geographic domain of interest and can therefore be very useful in determining exposures for people in areas not represented by AQS pollution monitors. It is important to assess the impact these metrics have on risk assessment estimation in the environmental health settings, as they can lead to the analysis of populations of people who have previously been excluded from most typical analyses due to their large distances from a nearby monitor. Comparing the health effect estimation results from use of these metrics with respect to the AQS data results has not been considered previously in this setting but can potentially have a large impact on the direction of future research.

For each exposure dataset, we fit the continuous pollution exposure model, and critical weeks during the pregnancy are analyzed. We compare the results from each metric, and the similarities and differences between the identified critical periods of the pregnancy are discussed. Based on these results, we extend the dataset to incorporate women who did not reside near an active pollution monitor within the region through use of the DS output, which includes ozone estimates across the region in space and time from 2001–2004. Susceptible periods during the entire pregnancy are then analyzed using the full birth dataset from this region.

In Section 2, we discuss the data used in the analysis, the statistical model, and the application method. The results and discussion are presented in Section 3. We close in Section 4 with the conclusions.

## 2. Materials and Methods

### 2.1. Data Description

**Figure 1:**
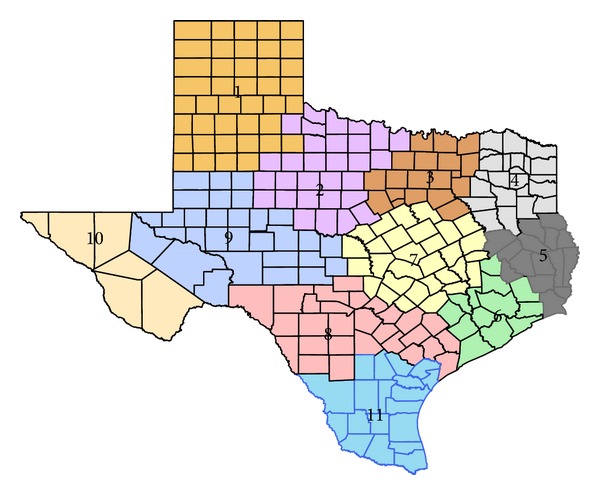
Texas Department of State Health Services health service region map. Health service region 11 is considered in the analysis.

 We analyze a dataset of full birth records from all births that occurred in the Texas Department of State Health Services (TDSHS) health service region 11, 2001–2004 (Figure 1). Adverse effects of ambient pollution exposure were recently investigated in this domain with respect to common cardiac congenital anomalies by Warren et al. [17]. In the analysis, we include full term births with completed gestational weeks between 37 and 44, inclusively. Each pregnancy must have also resulted in a live, singleton birth. Women with missing demographic covariates such as education level, age, and race/ethnicity are excluded from the analysis. The data were geocoded to the residence at delivery by the Geographic Information System group at the TDSHS. In total, we have 37,331 women with complete information in the region while 1,264 of these women lived within 8.49 kilometers (km) of an ozone monitor which was active daily during their pregnancy. We consider 8.49 km based on the layout of the 12 km × 12 km CMAQ grid. Further discussion is presented in Section 2.3.

The Air Quality System (AQS) monitoring data are obtained for the maximum daily 8-hour average ozone pollutant (parts per million (ppm)) in Texas from 2001 to 2004. The maximum daily 8-hour average values are used to determine the attainment status of nationally recognized standards. The AQS compiles air pollution data from multiple sources/monitors across the US and the data are used by many agencies, local and federal, to make air quality decisions (http://www.epa.gov/ttn/airs/airsaqs/basic_info.htm). There were 78 active monitors located in Texas from 2001 to 2004 with 6 located in TDSHS health service region 11.

The Community Multiscale Air Quality (CMAQ) deterministic chemistry model output is available for the maximum daily 8-hour average ozone pollutant (ppm) in 2001–2004 across Texas. The ozone estimates are provided daily on a 12 km × 12 km grid over Texas with each estimate representing the grid average. CMAQ output is provided in areas and times where observed pollution data are not directly available and allow for researchers to analyze the pollution composition in these areas. The CMAQ model is able to provide pollution estimates in geographic areas lacking sufficient monitoring data by relying on scientific expertise in air quality modeling and atmospheric science. CMAQ output is currently used for research and regulatory purposes across the country (http://ie.unc.edu/cempd/products/cmaq/overview.cfm). There were 4,786 grid point estimates located in Texas from 2001–2004 with 449 located in TDSHS health service region 11.

The Environmental Protection Agency (EPA) sponsored downscaler (DS) pollution output was developed by Berrocal et al. [18] and is publicly available at http://www.epa.gov/esd/land-sci/lcb/lcb_faqsd.html. The DS ozone estimates are provided daily from 2001 to 2004 (though not in all areas in 2001) at the census tract level and represent the 8-hour daily maximum ambient concentration (ppm) at that point. The process used to create the estimates relies on a spatial-temporal statistical model which combines the CMAQ output and AQS data to form estimates of the AQS data in areas where the data are not collected. The DS model is presented in a regression framework where the AQS observation is regressed on the closest CMAQ grid observation. The usual regression intercept and slope parameters are allowed to vary spatially and temporally to provide more flexible estimation across a domain of interest. The AQS observations are then predicted across the domain given the closest CMAQ value to the location of interest. There are known errors in the CMAQ data due to initial and boundary conditions, model parameterizations, mathematical approximations, and in the inputs that propagate through the model. The DS statistical model sets out to correct these errors through calibration of the CMAQ output while providing full spatial-temporal coverage over the US. Like the CMAQ output, the DS model output is provided in areas and times where observed pollution data are not directly available, making it very useful for pollution researchers. There were 4,388 DS locations in Texas from 2001 to 2004 with 341 located in TDSHS health service region 11.

### 2.2. Statistical Model

 We model low birth weight as a binary variable taking a value of one if birth *i* resulted in a birth weight of less than 2,500 g and zero otherwise. Conditional on the included model parameters, we assume independence between births such that $Y_{i}|p_{i}\overset{\text{ind}}{\sim }\text{Bernoulli}\left(p_{i}\right)$. The probability that pregnancy *i* results in low birth weight is given as

(1) Φ−1(pi)=xiTβ+∑j=1gaizj{ti(j),si}θj, i=1,…,n,

where Φ^−1^(·) is the inverse cumulative distribution function of the standard normal distribution, *ga*_*i*_ is the gestational age in completed weeks of person *i*, and *n* is the analysis sample size. The form of the function in (1) was shown to be successful in identifying critical windows of pollution exposure in terms of preterm birth by Warren et al. [16].

The statistical model is presented within the Bayesian framework. Bayesian methods differ from classical methods by treating the unknown model parameters as random quantities as opposed to fixed values. Before data are collected, a prior distribution is placed on the parameters which reflects the prior knowledge regarding the possible values of the parameters. After data collection, our prior beliefs are updated through use of the posterior distribution.

The posterior distribution is based on Bayes' Theorem [19] which, in terms of probability distribution functions, is given as *f*(***λ*** | **Y**) = *f*(**Y** | ***λ***)*f*(***λ***)/*f*(**Y**), where ***λ*** is the vector of unknown model parameters, **Y** is the vector of responses from the women in the analysis, *f*(**Y** | ***λ***) is the likelihood of the data based on independent Bernoulli responses, *f*(***λ***) is the prior distribution for the model parameters, and *f*(**Y**) is the marginal distribution of the observed data. The theorem implies that the most current information regarding the unknown parameters (given the data) is proportional to the usual likelihood multiplied by the prior distribution. Inference for the unknown parameters is conducted by summarizing the posterior distribution with the use of basic descriptive measures such as the mean, median, standard deviation, and distribution quantiles. If there is a lack of overall prior information regarding a model parameter, a vague prior distribution can be placed on the parameter and results obtained from the model fit will often closely match results from a classical statistical analysis. We utilize this method for the hyperparameters introduced in the model specification.

We prefer the Bayesian setting in the analysis due to the efficient algorithms that are available for fitting the model. The hierarchical nature of our model lends well to the use of Bayesian methods over the classical methods. The model fitting process is simplified through use of these Bayesian sampling techniques, while the results closely resemble those of a classical analysis through the use of vague prior distributions.

The **β** parameters are the coefficients that relate the covariates of interest to the probability of low birth weight. The x_*i*_ vector contains birth specific covariate information including the completed number of gestational weeks, year of birth, season of birth, average temperature in the state from the date of birth, average dewpoint in the state from the date of birth, maternal age group, education level, and race/ethnicity. The age groups in the analysis include 10–19, 20–24, 25–29, 30–34, 35–39, and 40+. The considered education levels are less than high school, high school, and greater than high school education. The racial/ethnic groups in the analysis are non-Hispanic black, non-Hispanic white, non-Hispanic other, and Hispanic. We model the average temperature and dewpoint measurements using a cubic B-spline with four degrees of freedom.

The *z*{*t*_*i*_(*j*), **s**_*i*_} terms represent the average ambient ozone amount at location **s**_*i*_ on calendar week *t*_*i*_(*j*), corresponding to the week of pregnancy for woman *i*. We allow the number of weeks of experienced pollution to vary for each woman, up to the point of the gestational age of the birth, in order to prevent exposure occurring after the birth of the child to affect the resulting probability of low birth weight.

The association between the experienced pollution and the probability of low birth weight is described through the ***θ*** = (*θ*_1_,…,*θ*_44_)^*T*^ parameters. In a typical statistical analysis, introducing an effect for each week of pregnancy in a multiple probit regression analysis leads to multicollinearity due to the correlation seen in the weekly averages. This multicollinearity causes the standard errors associated with the parameter estimates to be inflated and allows for the possibility that the signs of the parameter estimates could be incorrect. We avoid this issue by introducing a prior distribution for the ***θ*** vector which allows the effects to be correlated through the weeks thereby controlling the inflation of the standard errors and allowing for proper estimation of the effects. The prior is specified such that ***θ*** ~ MVN(0, *σ*_*θ*_^2^Σ(*ϕ*)), where Σ(*ϕ*)_*ij*_ = Corr(*θ*_*i*_, *θ*_*j*_) = exp⁡{−*ϕ*|*i* − *j*|}. This exponential structure allows effects of exposures that are separated by only a few weeks to be more highly correlated and for that correlation to decrease, as the number of weeks between the exposures increases.

We complete the model specification by assigning prior distribution for the model parameters. The **β** parameters are given independent normal distributions with large, fixed prior variance. The *ϕ* parameter, which controls the temporal smoothness of the ***θ*** parameters, is given a Uniform(0.0001,3) prior distribution. The overall variance parameter, *σ*_*θ*_^2^, is given an Inverse Gamma(3,2) prior distribution which leads to conjugacy in the model.

### 2.3. Application Preparation

We begin the analysis by assigning ambient ozone air pollution exposure amounts for each week during the pregnancy for every permissible birth in TDSHS health service region 11. We use the residence at delivery as the location of interest and match these locations with the closest active AQS pollution monitor on each day of the pregnancy. A number of studies have investigated the issue of maternal mobility during the pregnancy [20, 21]. These studies suggest that a majority of women do not move during the pregnancy, and those who do move only travel relatively short distances. Therefore, the introduced misclassification error is likely small.

For this initial analysis, births where there were no active monitors within 8.49 km (5.28 miles) of the residence at delivery, for any of the days during the pregnancy, are excluded from the analysis. We choose this distance because it corresponds to the maximum distance seen when working on a 12 km × 12 km grid (CMAQ) and also represents a common distance used in previous studies [22]. Once we create these daily exposure amounts, we average the daily readings to obtain weekly pregnancy averages.

Next, we repeat this process using the CMAQ and DS pollution output. Each residence at delivery in the dataset has a CMAQ and DS reading within 8.49 km due to the complete spatial coverage of these metrics. As a result, none of the women are removed as due to the lack of daily pollution information during the pregnancy. Once this dataset is complete, we create a subset of the data, such that it includes only the women who are also included in the AQS dataset. Therefore, these datasets allow for direct comparison with the AQS dataset since all included information is identical other than the pollution exposure amounts. These datasets allow us to compare the health effect estimation results of the CMAQ and DS output across the region with respect to the AQS monitoring data results.

## 3. Results and Discussion

 All results are presented for models fit in the Bayesian setting and based on 50,000 samples from the posterior distribution of each parameter of interest after a burnin period of 50,000 iterations. The fitting of each model is carried out using the R statistical software package [23].

### 3.1. Pollution Metric Analysis

 For the initial analysis, each exposure metric dataset contains 1,264 births across TDSHS health service region 11. About 2.3% of these full term births were low birth weight. We fit the model in (1), allowing for the pollution exposure estimates to change based on use of the AQS, CMAQ, and DS metrics. We modify (1), such that only the first 43 weeks of pregnancy are considered. Due to the smaller sample size in the region, we do not observe any births with a gestational age of 44 weeks and therefore cannot estimate *θ*_44_ in (1).

**Figure 2:**
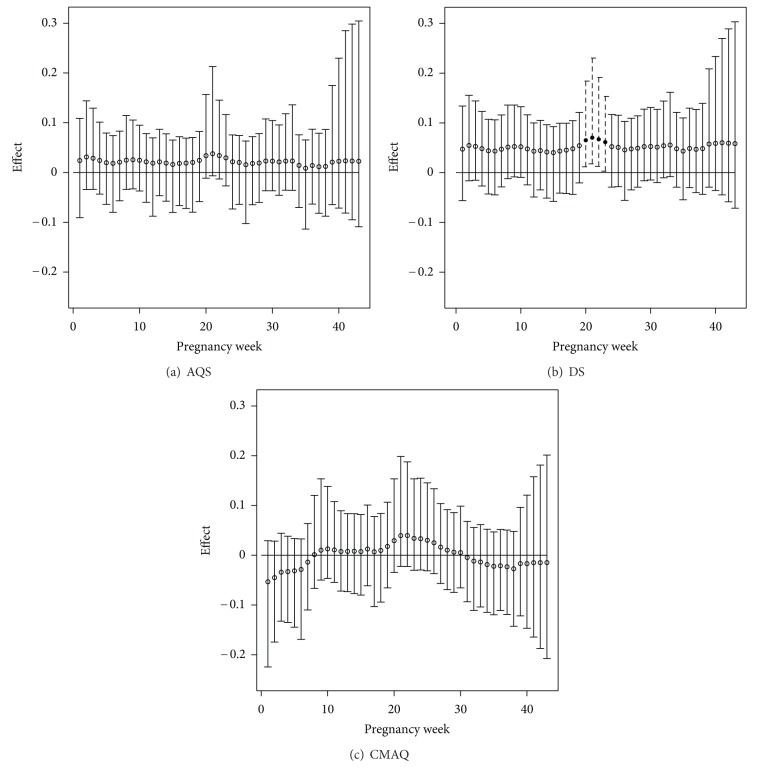
Susceptible windows of exposure results from the model in (1) using the AQS, DS, and CMAQ exposure metrics. Posterior medians and 95% credible intervals are displayed. Significant effects are shown with dotted lines and solid circles.

Figure 2 displays the graphical results from the model fit with each of the metrics. Each displayed effect represents the increase or decrease in *z*-score given a one standard deviation increase in the standardized pollution exposure during the relevant week of pregnancy. A significantly increased/decreased *z*-score leads directly to a significantly increased/decreased probability of low birth weight. Posterior medians and 95% credible intervals are displayed. Each analyzed dataset represents the same group of women and covariate information, with only the exposure amounts differing due to using the different exposure estimates.

The CMAQ and DS results differ from each other in terms of statistical significance and also from the AQS results. We can, however, see a similar trend in the estimated weekly effects across all metrics, with the weeks 19–25 appearing to be elevated in each of the plots. The DS risk assessment results agree much more with the AQS results overall while also identifying statistically significant effects in weeks 20–23 of pregnancy. Based on the DS results, there is sufficient evidence to suggest that increased ozone exposure during weeks 20–23 of pregnancy significantly increase the probability of low birth weight. The average length of the presented credible intervals for the model fit with each metric is 0.175 (AQS), 0.173 (DS), and 0.194 (CMAQ). The variability associated with the CMAQ risk assessment results is increased compared to the results of the other metrics. The CMAQ results are also less similar to the AQS results overall.

A possible explanation for these observed results is that the DS output is on average much more spatially dense across heavily populated regions of Texas than the AQS active monitors as well as the CMAQ 12 km × 12 km grid points.  Table 1 displays the summary statistics regarding the distance from a pollution estimate (AQS: active monitor; CMAQ: grid point; DS: census tract) for all women in Texas from 2001 to 2004 for each pollution metric. It is clear that the DS model output provides exposure estimates much closer to the women's residences in Texas than the AQS active monitors. As a result, we expect the DS and AQS results to be very similar, with a clearer signal shown in the DS output.

### 3.2. Extended DS Model Results

 As a result of the findings in Section 3.1, we are able to extend the analysis to include all available births in TDSHS health service region 11 from 2001–2004, not only those who lived close to an active AQS pollution monitor during their pregnancy. We are now able to include these women since the DS estimates are available daily over the entire state and appear to estimate the risk assessment similarly as the AQS data as well as provide shorter credible intervals on average than the uncalibrated CMAQ metric. Therefore, no woman is excluded from the analysis due to lack of ambient ozone data near her residence at delivery. As a result, our sample size is increased from 1,264 to 37,331.

Table 2 displays the included covariate results from the model in (1) using the DS weekly ozone exposures for all women in the region. These results suggest that there are a number of important factors which help to determine low birth weight status.

Giving birth in the fall season, with respect to winter, appears to significantly increase the probability of low birth weight for the child. Female babies are more likely to be born low birth weight, and as gestational age increases, the probability of low birth weight decreases. Black mothers (non-Hispanic) appear to have a higher probability of having a child with low birth weight than white mothers (non-Hispanic). As education level increases, the probability of low birth weight of the child significantly decreases. This effect is also true for the number of previous live births by a woman in the analysis.

Along with statistical model in (1), we also present results from two competing models. The presented models are as follows:

- Model 1: Bayesian probit regression model in (1) for low birth weight with weekly pollution exposure effects and prior temporal correlation structure assigned to the resulting parameters;
- Model 2: the standard trimester average multiple probit regression model where pollution exposures from each averaged trimester of pregnancy are input as covariates of interest;
- Model 3: multiple probit regression model where weekly exposures are included jointly, and multicollinearity is ignored.

All models control for the same maternal/seasonal covariates of interest. Model 2 represents the model most often found in the statistical epidemiologic literature where the risk of low birth weight is described by the exposures experienced during each trimester of pregnancy. Model 3 represents a naive attempt at jointly estimating the weekly effects and serves as a baseline to show what is gained by considering models 1 and 2.

**Figure 3:**
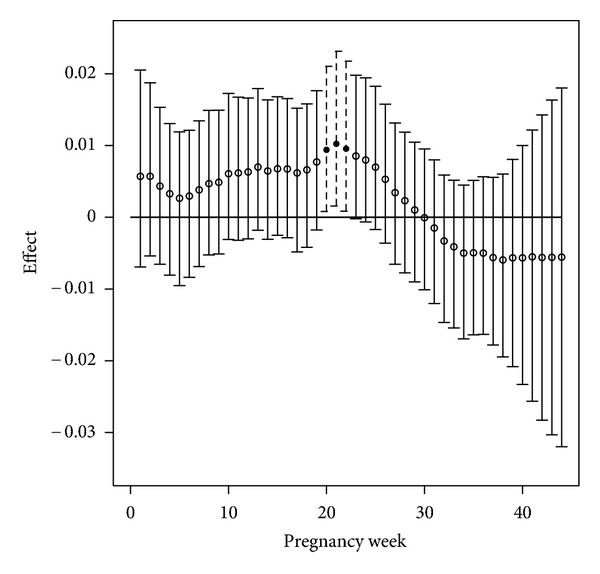
Susceptible windows of exposure results from the model in (1) using the DS weekly exposures for all women in TDSHS health service region 11. Posterior medians and 95% credible intervals are displayed. Significant effects are shown with dotted lines and solid circles.

**Figure 4:**
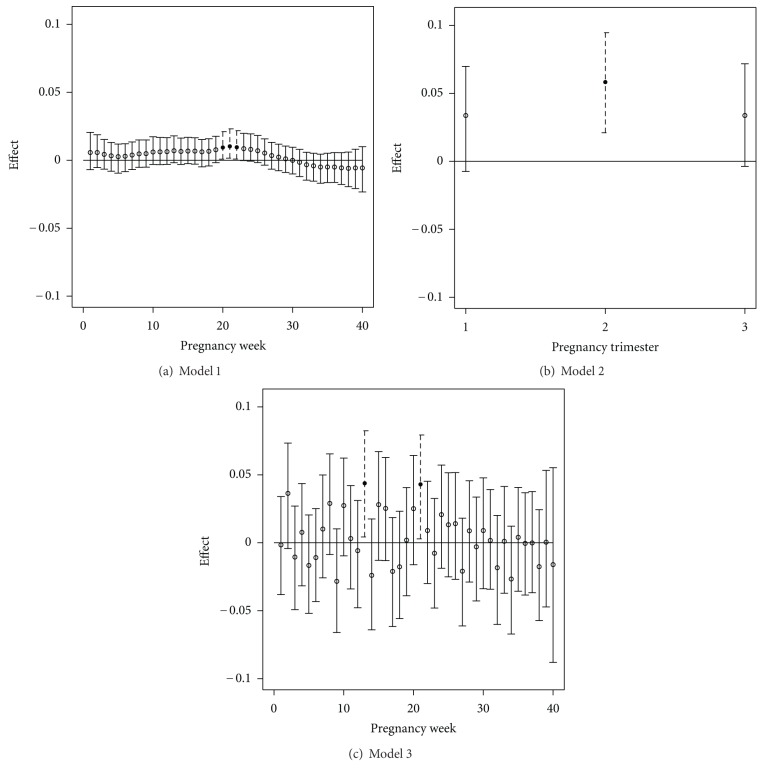
Susceptible windows of exposure results from models 1, 2, and 3 using the DS weekly exposures for all women in TDSHS health service region 11. Posterior medians and 95% credible intervals are displayed. Significant effects are shown with dotted lines and solid circles.

Figure 3 shows the graphical results from Model 1 using the extended DS dataset.  Figure 4 shows the graphical results for all models, displayed on the same scale for comparison purposes. The results from Model 2 suggest that an increased exposure to ozone during the second trimester significantly increases the probability of low birth weight for a woman residing in the region during 2001–2004. No statistically significant effects are seen in trimesters one and three. The Model 1 results, however, have the ability to more accurately describe this susceptible period in terms of weekly effects. We can now see that weeks 20–22 actually significantly increase the probability of low birth weight, as ambient ozone exposure increases. These significant results are shown more clearly in Figure 3. Identification of these weeks is also missed by Model 2 which can only detect trimester effects.

Simply entering weekly effects into a multiple probit regression setting (Model 3) does not produce reasonable results in terms of critical window identification. The multicollinearity associated with the weekly exposures makes it impossible to differentiate true signal from noise in the Model 3 results. The uncertainty associated with the parameter estimates is greatly increased due to the multicollinearity issue when compared with the Model 1 results. As a result, we only display the estimates for the first 40 weeks for Model 3 and Model 1, since the uncertainty associated with the final four weeks of pregnancy in Model 3 is extremely large.

When compared with Figure 2, the Model 1 results of Figure 3 are very similar in terms of identified critical windows. Due to the increased sample size introduced by extending to all women in the region, we obtain more precise estimates of the true effects. This can be seen by observing that the lengths of the credible intervals in Figure 3 are significantly shorter than those in Figure 2.

We use the deviance information criterion (DIC) in the Bayesian setting to describe model fit. The DIC is useful in comparing competing hierarchical models based on their overall fit and complexity, with smaller values indicating a better model [24]. The effective number of parameters (*p*_*D*_) describes the number of parameters being used in a particular model, helping to give insight to the model complexity. The DIC for Model 2 is 7,343.33 (*p*_*D*_ = 31.56), while for Model 1 it is 7,347.08 (*p*_*D*_ = 35.06). Differences of more than seven suggest that the model with smaller DIC is preferred. For reference, the DIC for Model 3 is 7,395.42 (*p*_*D*_ = 71.97), which shows its complete failure to efficiently model low birth weight. Models 1 and 2 have essentially the same DIC and *p*_*D*_ values, but it is clear from Figures 3 and 4 that Model 1 is preferred due to its ability to more accurately identify the susceptible periods of exposure.

## 4. Conclusions

 The model utilized in this paper allows us to specifically identify time periods during the pregnancy where increased exposure to ambient ozone adversely affects the resulting physical development of the child in terms of low birth weight. Low birth weight is known to lead to adverse and long-lasting health effects. Weeks late in the second trimester of pregnancy appear to be the most impactful with respect to the association between low birth weight and ambient ozone exposures. A number of alternative pollution metrics are available which attempt to fill in the spatial and temporal domains across a region for many common pollutants. We consider the CMAQ and DS products in the analysis and, based on our results, conclude that the DS model more closely resembles the AQS product with respect to health effect estimation and is therefore preferred in the low birth weight setting. The risk assessment estimates associated with the CMAQ metric have longer credible intervals on average and less resemble the AQS results. These results suggest that in the environmental health setting, calibrating the CMAQ output is important for future studies if working with a pollutant well represented by AQS data. This calibrated CMAQ output then gives similar results to the AQS data while minimizing variability in those results. The full spatial-temporal coverage of the CMAQ output is what allows the DS model to provide this calibrated output across the US.

Based on these findings, we extend the analysis to the entire TDSHS health service region 11 in Texas from 2001 to 2004 and identify the critical pregnancy weeks for birth weight. A clearer picture of the association between ambient ozone levels and low birth weight emerges as a result of the full spatial-temporal coverage of the DS output. Use of the metric allows us to include women who live in isolated parts of the region where ozone levels are not monitored while representing results we would expect to see if pollution monitors were located in these areas. These results would not be possible working with the AQS data alone. Based on the similarities with the AQS data results, the decreased credible interval lengths, and the excellent spatial-temporal coverage, we recommend use of the DS model output (http://www.epa.gov/esd/land-sci/lcb/lcb_faqsd.html) in epidemiologic models where AQS monitoring data are not available or are limited. The calibrated CMAQ output provided by the DS product is important for future studies of populations in regions where little pollution information exists.

Overall, it does appear that increased ambient ozone pollution exposure during the pregnancy is associated with a significant increase in the probability of low birth weight for the child. Use of the probit regression model allows for a more detailed analysis of these time periods that is not possible when the standard epidemiologic models are used. The model performs similarly to the standard epidemiologic model in terms of model selection criterion but is preferred due to its ability to specifically identify the susceptible periods of the pregnancy. This research further strengthens the evidence of the link between air pollution exposure and adverse birth outcomes while extending the results for low birth weight.

## Figures and Tables

**Table 1:** Statistical summaries describing the distribution of distances (km) from the closest pollution estimate for each pollution data (AQS) and model output (CMAQ, DS) source.

Metric	Mean	SD	Percentiles		
			0.025	0.50	0.975
AQS	42.19	90.02	1.65	11.92	390.90
CMAQ	4.68	1.91	1.04	4.78	7.96
DS	1.67	2.21	0.17	0.95	8.06

**Table 2:** Included covariate results for the model in (1) using the DS weekly exposures for all women in TDSHS health service region 11.

Covariate	Mean	SD	Percentiles		
			0.025	0.50	0.975
Intercept**	7.263	0.790	5.656	7.284	8.763
Gestational age (weeks)**	−0.243	0.014	−0.270	−0.243	−0.216
Season of birth					
Spring versus winter	0.047	0.074	−0.098	0.046	0.191
Summer versus winter	0.006	0.082	−0.152	0.006	0.167
Fall versus winter**	0.127	0.056	0.020	0.127	0.240
Female versus male baby**	0.226	0.031	0.166	0.226	0.287
Previous live births					
One versus none**	−0.244	0.039	−0.320	−0.244	−0.168
≥Two versus none**	−0.344	0.044	−0.429	−0.343	−0.259
Maternal age group					
10–19 versus 30–34	0.053	0.061	−0.065	0.053	0.173
20–24 versus 30–34	0.023	0.049	−0.074	0.023	0.120
25–29 versus 30–34	−0.013	0.048	−0.108	−0.013	0.082
35–39 versus 30–34	0.038	0.067	−0.094	0.038	0.169
≥40 versus 30–34	0.144	0.121	−0.101	0.145	0.375
Maternal race					
Black versus white**	0.484	0.158	0.159	0.488	0.787
Hispanic versus white	0.067	0.058	−0.044	0.067	0.183
Other versus white	0.292	0.151	−0.014	0.295	0.576
Maternal education					
High school versus <high school**	−0.140	0.038	−0.214	−0.140	−0.064
>High school versus <high school**	−0.175	0.043	−0.259	−0.176	−0.091
Birth year					
2002 versus 2001	−0.109	0.482	−0.933	−0.152	0.958
2003 versus 2001	0.145	0.482	−0.673	0.100	1.212
2004 versus 2001	0.112	0.483	−0.709	0.067	1.184

The (**) items have 95% credible intervals which do not include zero. The MC error for the means ranged from 0.0003 to 0.0164 with an average value of 0.0031.
